# Cognitive frailty in relation to adverse health outcomes independent of multimorbidity: results from the China health and retirement longitudinal study

**DOI:** 10.18632/aging.104078

**Published:** 2020-11-18

**Authors:** Chen Chen, JuYoung Park, Chenkai Wu, QianLi Xue, George Agogo, Ling Han, Emiel O. Hoogendijk, Zuyun Liu, Zunyou Wu

**Affiliations:** 1 National Center for AIDS/STD Control and Prevention, Chinese Center for Disease Control and Prevention, Beijing, China; 2 National Institute of Environmental and Health, Chinese Center for Disease Control and Prevention, Beijing, China; 3 Florida Atlantic University, Phyllis and Harvey Sandler School of Social Work, Boca Raton, FL 33431, USA; 4 Global Health Research Center, Duke Kunshan University, Kunshan, Jiangsu, China; 5 Duke Global Health Institute, Duke University, Durham, NC 27710, USA; 6 Division of Geriatric Medicine and Gerontology and Center on Aging and Health, Johns Hopkins Medical Institute, Baltimore, MD 21224, USA; 7 Centers for Disease Control and Prevention (CDC), Village Market, Nairobi, Kenya; 8 Department of Internal Medicine, Yale School of Medicine, New Haven, CT 06510, USA; 9 Department of Epidemiology and Biostatistics, Amsterdam Public Health Research Institute, Amsterdam UMC-location VU University Medical Center, Amsterdam, Netherlands; 10 Department of Big Data in Health Science, School of Public Health and the Second Affiliated Hospital, Zhejiang University School of Medicine, Hangzhou, China

**Keywords:** older adults, cognitive frailty, disability, death, multimorbidity

## Abstract

Our objectives were to evaluate: 1) the associations of cognitive frailty with various health outcomes including disability, hospitalization, and death; 2) whether the associations differed by multimorbidity. We included data of 5113 Chinese older adults (aged 60+ years) who had baseline cognition and physical frailty assessments (2011 wave) and follow-up for 4 years. About 16.0% (n=820) had cognitive impairment; 6.7% (n=342) had physical frailty; and 1.6% (n=82) met criteria for cognitive frailty. Both cognitive impairment (odds ratios (ORs) range: 1.41 to 2.11) and physical frailty (ORs range: 1.51 to 2.43) were independently associated with basic activities of daily living (BADL), instrumental ADL (IADL), mobility disability, hospitalization, and death among participants without that corresponding outcome at baseline, even after accounting for covariates. Relative to participants who had normal cognition and were nonfrail, those with cognitive frailty had the highest risk for IADL disability (OR=3.40, 95% CI, 1.23–9.40) and death (OR=3.89, 95% CI, 2.25–6.47). We did not find significant interaction effects between cognitive frailty and multimorbidity (P_interactions_>0.05). Overall, cognitive frailty was associated with disability and death, independent of multimorbidity. This highlights the importance of assessing cognitive frailty in the community to promote primary and secondary preventions for healthy aging.

## INTRODUCTION

Both cognitive impairment and physical frailty are important indicators of the aging process, and are associated with adverse health outcomes including disability, hospitalization, and death [1, 2]. Due to the potential interaction between these two aging indicators, they have been incorporated together as a new concept, cognitive frailty, defined as the simultaneous presence of both cognitive impairment and physical frailty in non-demented older adults, proposed in 2013 by an (I.A.N.A./I.A.G.G.) international consensus group [3]. Cognitive frailty describes a preclinical cognitive status caused by physical frailty instead of neurodegenerative disorders [4, 5]. The fundamental hypothesis about cognitive frailty is the direction of causality between physical frailty and cognitive impairment, i.e. physical frailty precedes and causes cognitive impairment [4]. Cognitive frailty is different from the frailty index (FI), which is based on the degree of accumulation of health deficits and represents an alternative instrument of frailty that incorporates more health dimensions, including comorbidities, psychological factors, symptoms, and disabilities [6]. However, the FI dose not underline the temporal relationship between physical frailty and cognitive impairment. So the main benefit of the construction of cognitive frailty is to explain heterogeneity in etiology of cognitive impairment so as to improve intervention target.

A few studies have reported that cognitive frailty is more strongly associated with adverse health outcomes, such as disability in basic activities of daily living (BADL) [7–9], low quality of life [9], and death [8–14], than cognitive impairment or physical frailty alone [7–10, 15]. Nearly all of these studies were conducted in developed countries, with one exception (in China) [14], which is limited in study setting (e.g., one province) and small in sample size [14] (For detailed information see Supplementary Table 1). To date, little is known about whether cognitive impairment and physical frailty jointly associate with adverse outcomes in nationwide samples of older adults from developing countries like China. The investigation of this topic in different countries is crucial due to differences in population characteristics (e.g., lifestyle factors, pattern of chronic diseases). Moreover, in the context of dramatic population aging in China, early screening and intervention of at-risk older adults [16] may result in substantial reductions in healthcare expenditures, one of the key challenges in the near future.

Early identification of high-risk older adults is challenging partly due to the complexity of health issues that one older adult has. One key facet of such complexity is multimorbidity, characterized as co-existence of multiple chronic diseases/conditions within one individual [17]. It has been reported that 42.4% of older adults in China have multimorbidity [18], which is associated with worse functioning [19], poor quality of life [19, 20], and high mortality [21]. Cognitive frailty has become a novel target for achieving successful ageing [22]; therefore, confirming whether cognitive frailty is associated with adverse health outcomes independent of multimorbidity is of great importance to prioritize potential preventive interventions; however, it has not been investigated in previous studies in both developed countries [7–13, 15] and in China [14].

To address these questions, we used data from the China Health and Retirement Longitudinal Study (CHARLS), a large cohort study with a nationally representative sample of middle-aged and older adults in China. The objective of this study was two-fold: first, to evaluate the associations of cognitive frailty with an array of health outcomes including disability (BADL, instrumental ADL [IADL], and mobility), hospitalization, and death; and second, to determine whether the associations differed by multimorbidity.

## RESULTS

### Characteristics of study participants

Among the 5113 participants at baseline, mean (SD) age was 68.1 (6.5) years, and 49% (n=2479) were women (Table 1). The majority of participants at baseline had less than six years of education (including no formal education, did not finish primary school and elementary school), were currently married, lived in rural areas, and were more likely to be non-smokers and non-drinkers.

**Table 1:** Baseline characteristics of the study participants, CHARLS 2011.

**Characteristic**	**Overall**	**Group 1 (Normal cognition & Nonfrail)**	**Group 2 (Cognitive impairment & Nonfrail)**	**Group 3 (Normal cognition & Frail)**	**Group 4 (Cognitive Frailty)**	**P-value**
	N=5113	N=4033	N=738	N=260	N=82	
Age, years^a^	68.1 ± 6.5	67.9 ± 6.3	67.3 ± 6.0	73.0 ± 7.9	70.8 ± 7.4	<0.01
60-79 years old	4792 (93.7)	3804 (94.3)	714 (96.8)	205 (78.9)	69 (84.2)	<0.01
80+ years old	321 (6.3)	229 (5.7)	24 (3.3)	55 (21.2)	13 (15.9)	
Gender						<0.01
Women	2479 (48.5)	1758 (43.6)	541 (73.3)	123 (47.3)	57 (69.5)	
Men	2634 (51.5)	2275 (56.4)	197 (26.7)	137 (52.7)	25 (30.5)	
Education						<0.01
No formal education	1812 (35.4)	1077 (26.7)	550 (74.5)	117 (45.0)	68 (82.9)	
Did not finish primary school	1091 (21.3)	896 (22.2)	126 (17.1)	64 (24.6)	5 (6.1)	
Elementary school	1290 (25.2)	1179 (29.2)	51 (6.9)	52 (20.0)	8 (9.8)	
Middle school	614 (12.0)	578 (14.3)	9 (1.2)	26 (10.0)	1 (1.2)	
High school or above	306 (6.0)	303 (7.5)	2 (0.3)	1 (0.4)	0 (0)	
Marital status						<0.01
Currently married	4056 (79.3)	3274 (81.2)	559 (75.8)	175 (67.3)	48 (58.5)	
Others	1057 (20.7)	759 (18.8)	179 (24.3)	85 (32.7)	34 (41.5)	
Residence						<0.01
Rural	3233 (63.2)	2404 (59.6)	588 (79.7)	178 (68.5)	63 (76.8)	
Urban	1880 (36.8)	1629 (40.4)	150 (20.3)	82 (31.5)	19 (23.2)	
Smoking status						<0.01
Nonsmoker	2886 (56.5)	2152 (53.4)	545 (73.9)	137 (52.7)	52 (63.4)	
Ever smoker	623 (12.2)	549 (13.6)	34 (4.6)	35 (13.5)	5 (6.1)	
Smoker	1603 (31.4)	1331 (33.0)	159 (21.5)	88 (33.9)	25 (30.5)	
Alcohol consumption						<0.01
No	3019 (59.1)	2271 (56.4)	520 (70.6)	168 (64.6)	60 (73.2)	
Yes	2090 (40.9)	1759 (43.7)	217 (29.4)	92 (35.4)	22 (26.8)	
Body mass index^a^	22.9 ± 3.9	23.2 ± 3.9	22.4 ± 3.8	21.7 ± 4.7	21.4 ± 4.1	<0.01
Number of chronic diseases	1.5 ± 1.3	1.5 ± 1.3	1.4 ± 1.3	2.0 ± 1.4	1.5 ± 1.2	<0.01
Multimorbidity						<0.01
No	2856 (55.9)	2267 (56.2)	437 (59.2)	106 (40.8)	46 (56.1)	
Yes	2257 (44.1)	1766 (43.8)	301 (40.8)	154 (59.2)	36 (43.9)	

CHARLS, the China Health and Retirement Longitudinal Study.

Values are given as No. (percentages). ^a^ Values are given as Mean ± SD.

Percentages summed up by column, which may not sum to 100 because of rounding. There were missing data on smoking status (n=1), alcohol consumption (n=4), and body mass index (n=11).

At baseline, about 16.0% (n=820) of all participants had cognitive impairment; 6.7% (n=342) had physical frailty; and 1.6% (n=82) had both components, i.e., met criteria for cognitive frailty. As expected, the oldest-old (aged 80+ years) were more likely to have normal cognition and physical frailty (Group 3) or cognitive frailty (Group 4). A higher proportion of women were found in the nonfrail group with cognitive impairment (Group 2) and in the group with cognitive frailty (Group 4), which mainly represent older adults with cognitive impairment. About 44% of all participants had two or more chronic diseases (i.e., multimorbidity). Similarly, about 44% of participants in Group 4 had multimorbidity. A little higher proportion of participants in Group 3 (59%) had multimorbidity. Overall, we did not find obvious differences in the proportion of multimorbidity between the four cognitive frailty groups.

### Associations between cognitive frailty and adverse health outcomes

### BADL, IADL, and mobility disability

Among the participants without BADL disability at baseline (n=3341), 33.6% (n=1121) reported BADL disability over 4-year follow-up. Similarly, the percentages for incident IADL and mobility disability were 35.2% (n=1136) and 65.3% (n=1101), respectively.

Table 2 shows the individual and combined associations of cognitive impairment and physical frailty with adverse health outcomes, using the normal cognition and nonfrail group (Group 1) as the reference group. After adjusting for age and gender, both cognitive impairment and physical frailty significantly increased the risk of developing BADL, IADL, and mobility disability, with ORs ranging from 1.41 (95% CI: 1.15–1.74) to 2.43 (95% CI: 1.64–3.62). In combined effect analyses, belonging to Group 4 (cognitive frailty) was strongly associated with an increased risk for BADL and IADL disability, with ORs of 2.09 and 3.40, respectively, although the OR for BADL was not statistically significant, due to the small sample size for Group 4. Only four participants were free of mobility disability at baseline, which all developed mobility disability during the follow-up period.

**Table 2:** Associations of cognitive impairment and physical frailty with disability (BADL, IADL, and mobility), hospitalization, and death in full sample, CHARLS 2011-2015.

		**Model 1**	**Model 2**
	**No. of events/No. of participants**	**OR (95% CI)**	**OR (95% CI)**
**BADL disability**			
**Total**	1121/3341		
**Individual effect**			
Cognitive impairment			
Normal cognition	924/2867	Ref.	Ref.
Cognitive impairment	197/474	1.41 (1.15–1.74)	1.38 (1.10–1.73)
Physical Frailty			
Nonfrail	1042/3207	Ref.	Ref.
Frail	79/134	2.38 (1.66–3.43)	2.16 (1.49–3.13)
**Combined effect**			
Group 1**(**Normal cognition & Nonfrail)	859/2758	Ref.	Ref.
Group 2**(**Cognitive impairment & Nonfrail)	183/449	1.43 (1.16–1.77)	1.40 (1.11–1.76)
Group 3**(**Normal cognition & Frail)	65/109	2.61 (1.74–3.90)	2.31 (1.53–3.48)
Group 4**(**Cognitive Frailty)	14/25	2.09 (0.93–4.71)	2.22 (0.97–5.08)
**IADL disability**			
**Total**	1136/3226		
**Individual effect**			
Cognitive impairment			
Normal cognition	924/2820	Ref.	Ref.
Cognitive impairment	212/406	2.11 (1.70–2.63)	1.79 (1.41–2.27)
Physical Frailty			
Nonfrail	1069/3114	Ref.	Ref.
Frail	67/112	2.43 (1.64–3.62)	2.13 (1.42–3.21)
**Combined effect**			
Group 1**(**Normal cognition & Nonfrail)	868/2725	Ref.	Ref.
Group 2**(**Cognitive impairment & Nonfrail)	201/389	2.13 (1.71–2.67)	1.81 (1.42–2.30)
Group 3**(**Normal cognition & Frail)	56/95	2.56 (1.66–3.94)	2.23 (1.43–3.47)
Group 4**(**Cognitive Frailty)	11/17	3.40 (1.23–9.40)	2.80 (1.00–7.87)
**Mobility disability**			
**Total**	1101/1685		
**Individual effect**			
Cognitive impairment			
Normal cognition	959/1499	Ref.	Ref.
Cognitive impairment	142/186	1.53 (1.06–2.21)	1.62 (1.09–2.40)
Physical Frailty			
Nonfrail	1075/1652	Ref.	Ref.
Frail	26/33	1.51 (0.64–3.59)	1.47 (0.61–3.53)
**Combined effect**			
Group 1**(**Normal cognition & Nonfrail)	937/1470	Ref.	Ref.
Group 2**(**Cognitive impairment & Nonfrail)	138/182	1.51 (1.04–2.18)	1.58 (1.06–2.36)
Group 3**(**Normal cognition & Frail)	22/29	1.38 (0.57–3.33)	1.29 (0.52–3.18)
Group 4**(**Cognitive Frailty)	4/4	1*	1*
**Hospitalization**			
**Total**	1075/3776		
Cognitive impairment			
Normal cognition	891/3164	Ref.	Ref.
Cognitive impairment	184/612	1.14 (0.94–1.39)	1.26 (1.02–1.56)
Physical Frailty			
Nonfrail	994/3561	Ref.	Ref.
Frail	81/215	1.37 (1.02–1.83)	1.34 (0.99–1.80)
**Combined effect**			
Group 1**(**Normal cognition & Nonfrail)	825/2997	Ref.	Ref.
Group 2**(**Cognitive impairment & Nonfrail)	169/564	1.17 (0.96–1.44)	1.29 (1.04–1.62)
Group 3**(**Normal cognition & Frail)	66/167	1.50 (1.08–2.07)	1.43 (1.03–2.00)
Group 4**(**Cognitive Frailty)	15/48	1.11 (0.60–2.08)	1.29 (0.68–2.44)
**Death**			
**Total**	434/5113		
**Individual effect**			
Cognitive impairment			
Normal cognition	353/4293	Ref.	Ref.
Cognitive impairment	81/820	1.58 (1.21–2.08)	1.49 (1.11–2.00)
Physical Frailty			
Nonfrail	363/4771	Ref.	Ref.
Frail	71/342	2.07 (1.53–2.81)	1.82 (1.33–2.48)
**Combined effect**			
Group 1**(**Normal cognition & Nonfrail)	303/4033	Ref.	Ref.
Group 2**(**Cognitive impairment & Nonfrail)	60/738	1.42 (1.05–1.92)	1.34 (0.97–1.85)
Group 3**(**Normal cognition & Frail)	50/260	1.82 (1.28–2.58)	1.59 (1.11–2.28)
Group 4**(**Cognitive Frailty)	21/82	3.89 (2.25–6.74)	3.49 (2.00–6.15)

CHARLS, the China Health and Retirement Longitudinal Study; BADL, basic activity of daily living; IADL, instrumental activity of daily living; OR, odds ratio; CI, confidence interval.

As described in Methods, we ran a logistic regression model for each health outcome (e.g., BADL disability) in participants who did not have exposure for that outcome at baseline (i.e., N=3341 participants without BADL disability at baseline). Odds ratios (ORs) and corresponding 95% confidence interval (CI) are presented. Model 1 adjusted for age and gender. Model 2 further adjusted for education, residence, marital status, smoking status, alcohol consumption, body mass index, and multimorbidity.

*Since all participants in this subgroup reported the health outcome over the follow-up period, we assigned 1 to them.

### Hospitalization

About 28.5% (n=1075) of the participants who did not report hospitalization at baseline reported a hospitalization during 4-year follow-up. In individual effect analyses, physical frailty (OR: 1.37, 95% CI: 1.02–1.83), but not cognitive impairment (OR: 1.14, 95% CI: 0.94–1.39), was independently associated with incidence of hospitalization. In combined effect analyses, compared with Group 1, the other three groups were all associated with elevated risk for hospitalization, though only Group 3 (Normal cognition and frail; OR: 1.50, 95% CI, 1.08‒2.07, Table 2) met statistical significance.

### Death

Over 4-year follow-up, 434 of 5113 (8.5%) participants died. In individual effect analyses, both cognitive impairment and physical frailty were independently associated with an increased risk of death (OR=1.58 and 2.07, respectively). In combined effect analyses, all groups were associated with elevated risk of death (ORs range from 1.42 to 3.89). Compared with Group 1, Group 4 (cognitive frailty) had a nearly four-fold increased risk of death (OR=3.89, 95% CI, 2.25‒6.74, Table 2),

For all (individual and combined) associations above, we did not observe substantial changes of the results when additionally adjusting for education, residence, marital status, smoking status, alcohol consumption, body mass index, and multimorbidity (Table 2, Model 2). Furthermore, additional analyses revealed that for the above individual effect analyses, mutually controlled associations of cognitive impairment and physical frailty with all outcomes in logistic regression models did not affect the main model results (Supplementary Table 2).

### Subgroup analyses by multimorbidity

We found that after adjusting for age and gender, multimorbidity significantly increased the risk of the five adverse health outcomes (Supplementary Table 3), which suggested that multimorbidity could play a role as moderator or confounder of the associations between cognitive frailty and adverse health outcomes.

Table 3 presents the associations of cognitive impairment and physical frailty with disability (ADL, IADL, and mobility), hospitalization, and death in older adults without and with multimorbidity. We did not find significant interaction effects between cognitive frailty and multimorbidity for these adverse outcomes (all interaction P values>0.05). As shown in Table 3, we found that in participants without multimorbidity, the risk estimates were similar to those shown in the full sample, where cognitive frailty was a strong predictor for the health outcomes (ORs range from 3.59 to 4.47) except for hospitalization. In participants with multimorbidity, partly due to the small sample size, we did not observe statistically significant associations of cognitive frailty with the health outcomes, but the overall trend suggested that older adults with cognitive frailty were at high risk of IADL disability and death.

**Table 3:** Associations of cognitive impairment and physical frailty with disability, hospitalization, and death in persons without or with multimorbidity, CHARLS 2011-2015.

	**BADL disability**	**IADL disability**	**Mobility disability**	**Hospitalization**	**Death**
**Without multimorbidity**	N=2011	N=1913	N=1176	N=2212	N=2856
Group 1	Ref.	Ref.	Ref.	Ref.	Ref.
Group 2	1.44 (1.09–1.91)	2.60 (1.96–3.45)	1.52 (1.02–2.26)	1.22 (0.93–1.60)	1.86 (1.27–2.73)
Group 3	2.53 (1.42–4.52)	2.62 (1.37–5.00)	1.35 (0.41–4.46)	1.33 (0.80–2.22)	1.42 (0.80–2.52)
Group 4	3.68 (1.38–9.80)	4.47 (1.28–15.58)	1*	1.33 (0.59–2.98)	3.59 (1.67–7.80)
**With multimorbidity**	N=1330	N=1313	N=509	N=1564	N=2257
Group 1	Ref.	Ref.	Ref.	Ref.	Ref.
Group 2	1.71 (1.21–2.42)	1.86 (1.28–2.72)	2.34 (0.80–6.92)	1.21 (0.89–1.65)	0.98 (0.58–1.63)
Group 3	2.31 (1.31–4.09)	2.13 (1.19–3.81)	1.04 (0.27–3.94)	1.42 (0.93–2.18)	1.99 (1.26–3.12)
Group 4	0.79 (0.17–3.60)	2.58 (0.44–15.01)	‒	1.91 (0.34–2.44)	4.34 (1.96–9.60)
**Interaction P value: Cognitive frailty × multimorbidity**	0.232	0.525	0.552	0.883	0.227

CHARLS, the China Health and Retirement Longitudinal Study; BADL, basic activity of daily living; IADL, instrumental activity of daily living; Group 1, Normal cognition & Nonfrail; Group 2, Cognitive impairment & Nonfrail; Group 3, Normal cognition & Frail; Group 4, Cognitive Frailty.

As described in Methods, we ran a logistic regression model for each health outcome (e.g., BADL disability) in participants who did not have exposure for that outcome at baseline (e.g., N=2011 participants without BADL disability at baseline and had no multimorbidity). Odds ratios (ORs) and corresponding 95% confidence interval (CI) are presented. All models were adjusted for age and gender.

*Since all participants in this subgroup reported the health outcome over the follow-up period, we assigned 1 to them.

### Sensitivity analyses

In sensitivity analyses, we found that: (1) the results were unchanged after removing 146 participants who reported having memory-related disease (dementia, Parkinson's disease, etc., Supplementary Table 4); (2) accounting for the competing risk of death did not substantially change the associations of cognitive frailty with disabilities and hospitalization appreciably (Supplementary Table 5); (3) the cross-sectional analyses obtained relatively higher risk estimates and further offered supportive evidence for the longitudinal relationships of cognitive impairment, physical frailty, and their combinations with adverse health outcomes (Supplementary Table 6).

## DISCUSSION

To facilitate early identification of high-risk older adults in China, we evaluated the association of one important new construct, cognitive frailty, with a series of adverse outcomes in a large sample from CHARLS, a cohort study of middle-aged and older Chinese adults. We found that older adults with cognitive frailty have the highest risks of BADL, IADL disability and death, relative to their counterparts who had normal cognition and were nonfrail, even after accounting for several covariates. Overall, these associations did not differ by multimorbidity. These findings suggest the possibility and importance of using cognitive frailty as a tool to help with risk stratification in the general Chinese older population.

In this sample of community-dwelling older adults, 1.6% met the criteria for cognitive frailty, in line with a recent literature review [5], which reported that the prevalence of cognitive frailty ranges from 1% to 5% in community-dwelling older adults worldwide. We acknowledge the influence of the exclusion of participants with missing data on cognition or physical frailty components, which is inevitable in large-scale epidemiological investigations. Nevertheless, this prevalence is remarkable when it comes to the very large population of older adults in China (i.e., 249 million aged 60+ years in 2018). That being said, approximately 3.9 million older adults may have cognitive frailty in China, and may require urgent and considerable attention.

Overall, our results of the combined associations of cognition and physical frailty with adverse outcomes were consistent with previous research (Supplementary Table 1), concerning death [7, 9, 10, 12–14, 23], BADL [7–9, 23], IADL [8, 9, 15, 23], mobility disability [8, 23], and hospitalization [8, 9, 14], although in the current study the estimated risk for cognitive frailty with regard to hospitalization was not statistically significant. These non-significant findings regarding hospitalization may reflect diverse pathways or risk factor profiles [23]. For example, hospitalization is often the result of acute illness or injury rather than the result of cognitive impairment or physical frailty itself.

To the best of our knowledge, this is the first study to explore the role of multimorbidity in the associations of cognitive frailty with various health outcomes in Chinese older adults. The comparable findings in subgroups with and without multimorbidity suggest that cognitive frailty and multimorbidity do not necessarily share the same underlying pathophysiologic pathways and mechanisms towards disability and death. Considering the complexity of aging, beyond the physical and cognitive dimensions (which cognitive frailty captures), multimorbidity per se could result from various factors such as nutrition [24] and mental health [25] (although these factors are also associated with cognitive frailty), and could lead to many adverse health outcomes. Finally, we acknowledge that the potential recall bias, mostly underreported, resulted from the self-report of multimorbidity, might introduce exposure misclassification on multimorbidity and might obscure the true estimated associations, although the estimated prevalence of multimorbidity (43.8%) in this study was consistent with previous studies based on CHARLS (42.4%) [18] and other Chinese cohorts (44.4%) [26].

The clinical utility and relevance of our findings should be placed in the context of the large older population in China and relatively limited healthcare resources. As mentioned above, about 3.9 million older adults may suffer from cognitive frailty. We have demonstrated that cognitive frailty, relative to cognitive impairment and physical frailty individually, could be a better measure of vulnerability because it is more sensitive and thus captures more susceptible older adults who were currently non-disabled but at high risk of developing disability (such as, BADL) or other subsequent adverse consequences. Early identification of older adults with cognitive frailty has public health implications, in both clinical practice and large epidemiological screening settings, considering the fact that there are still no effective pharmacological treatments to improve physical frailty status [27] and cognitive impairment [28]. Targeting this specific population would be a priority, especially when considering that interventions may be more effective if applied at an early stage. Nowadays, many comprehensive geriatric assessments are being done in China [29, 30], which include detailed physical examinations and offer an opportunity for the application of cognitive frailty. Since the assessment of cognitive frailty is not simple currently, appropriate implementation while conducting comprehensive geriatric assessment should be explored in the near future.

In the current study, data from a nationwide prospective cohort study in China and the availability of data on multiple health outcomes in a relatively large cohort provided a unique opportunity to evaluate the associations between cognition, physical frailty, and multiple adverse outcomes, of which some (e.g., IADL, mobility disability, and hospitalization) have not been investigated previously. Second, the high quality of the data, low rate of loss-to-follow-up, and comparable study design of the CHARLS (compared to other international Health and Retirement Study sister studies [31]) strengthen our findings. Future research could attempt to explore the associations of cognitive frailty and health outcomes in more countries and cohorts (e.g., UK BIOBANK).

The current study nevertheless has several limitations. First, we used a modified definition to construct the physical frailty phenotype compared with the one proposed by Fried (e.g., the use of a self-reported measure of activity intensity-whether walk 10 or more minutes continuously during a usual week-rather than the original kilocalories expended index), which would influence the proportion of physical frailty to some extent. However, the utility and validity of this definition for physical frailty in identifying frail Chinese adults have been previously demonstrated [32]. Second, the timing of the occurrence of health outcomes was not available in this study. Third, the exclusion of participants with missing data on physical frailty or cognition and/or the restriction to participants without outcome at baseline (in Table 2) would have induced selection bias and resulted in limited sample size for some outcomes (e.g., mobility disability). Yet, the effects of this bias are probably relatively small since we observed similar results across these health outcomes while the missingness was randomized to some extent. Fourth, although the overall sample size is relatively large, some group (such as Group 4, cognitive frailty) are still not large enough in subgroup analysis, which may lead to false positive results. At the same time, we observed a consistent trend, that is, from Group 1 to Group 4, the risks of outcomes are gradually increasing, which has little to do with the power itself. Finally, the study attrition (e.g., death, loss-to-follow-up) could be problematic in the longitudinal cohort. However, when running competing risk Cox regression models to account for the influence of death, we did not find substantial changes to the results (Supplementary Table 5).

## CONCLUSIONS

In a large sample of Chinese community-dwelling older adults, we demonstrated that overall, cognitive frailty was associated with disability and death, independent of multimorbidity. In the context of rapid population aging, these findings highlight the importance of assessing cognitive frailty in community to promote primary and secondary preventions for healthy aging.

## MATERIALS AND METHODS

### Study population

The CHARLS targeted Chinese community-dwelling adults aged 45 years and older and their spouses. The CHARLS used a multistage sampling strategy covering 28 provinces, 150 counties/districts, and 450 villages/urban communities across the country. Participants were first recruited in 2011/2012, and completed two follow-up visits biennially up to 2015/2016. Details of the CHARLS survey have been described elsewhere [31]. The CHARLS study offers a wide range of information on socioeconomic status and health, including demographic characteristics, family structure, health status and functioning, biomarkers, health care and insurance, work, retirement and pension, income and consumption, and assets.

**Figure 1:**
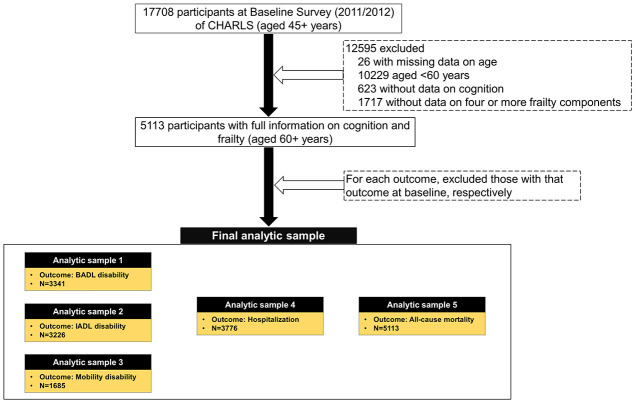
**Flow chart of analytic sample. CHARLS, the China Health and Retirement Longitudinal Study; BADL, basic activity of daily living; IADL, instrumental activity of daily living.**

Out of 17,708 participants aged 45 years and older enrolled in the baseline survey (2011/2012), we excluded those with missing data on age (n=26), aged <60 years (n=10229), with missing data on cognition (n=623) and four or more physical frailty components (n=1717), leaving 5113 participants available (Figure 1). Finally, for different adverse health outcomes, we assembled various sets of analytical samples, as shown below:

Sample 1 for BADL disability: restricted to participants without BADL disability at baseline (n=3341);

Sample 2 for IADL disability: restricted to participants without IADL disability at baseline (n=3226);

Sample 3 for mobility disability: restricted to participants without mobility disability at baseline (n=1685);

Sample 4 for hospitalization: restricted to participants without hospitalization occurred at baseline (n=3776);

Sample 5 for death: all participants (n=5113).

### Measures

### Cognitive impairment

Following prior CHARLS publications [33, 34], we used three assessments of cognition: the Telephone Interview of Cognitive Status (TICS-10), word recall, and figure drawing. The overall cognition score is the sum score of the TICS-10 (orientation and attention, 0-10), word recall (episodic memory, 0-10), and figure drawing (visual spatial abilities, 0-1). The TICS is a well-established measure for assessing mental status [35]. In CHARLS, ten items from TICS were included: date (month, day, and year), day of the week, season of the year, and serial subtraction of 7 from 100 (up to five times). Summing the number of correct answers to these questions resulted in a TICS-10 score, ranging from 0 to 10. TICS-10 primarily assesses the executive functions of orientation to time and attention [36]. For word recall, participants were asked to memorize and immediately recall as many words as they could in any order immediately after interviewers read a list of 10 Chinese nouns (i.e., immediate recall). Four to 10 minutes later, participants were asked to recall as many of the original words as possible (i.e., delayed recall) [34, 35]. The average number of immediate and delayed word recalls ranged from 0 to 10, and is regarded as assessing episodic memory [37]. For figure drawing, participants were shown a picture of two overlapped pentagons and asked to draw a similar figure. Participants who successfully completed the task received a score of 1, and those who failed received a score of 0. This task assessed visuospatial abilities [38]. A summary score of the three parts range from 0 to 21, with higher scores indicating better performance [33, 34]. According to the literature, participants were classified as having cognitive impairment if their summary score fell more than 1 SD below age-appropriate norms; otherwise, they were defined as normal cognition [39].

### Physical frailty

Frailty was measured by an adapted version of the Fried physical frailty phenotype approach [40], and has been previously developed and validated in the CHARLS [32]. Five items/criteria are included: shrinking, weakness, exhaustion, slowness, and inactivity. Shrinking was established as self-reported loss of 5 or more kg in the previous year or having a body mass index (BMI) of 18.5 kg/m^2^ or less. Weakness was defined as the lowest quintile on maximum handgrip strength (either hand; two trials for each; measured in a standing position with arm bended at 90 degrees) among the population, adjusting for gender and BMI. Exhaustion was determined according to two questions from the Center for Epidemiological Studies-Depression scale: “I could not get going” and “I felt everything I did was an effort”. Slowness was defined as the lowest quintile on the average of two-timed walk tests over a 2.5-meter course, at usual pace, among the population, adjusting for gender and standing height. Inactivity was determined if participants self-reported that they did not walk 10 or more minutes continuously during a usual week. We modified the original physical frailty phenotype proposed by Fried et al. [40], due to limited availability of data in CHARLS. Participants who met three or more criteria were defined as having physical frailty (frail); otherwise, they were considered as having no physical frailty (nonfrail).

### Cognitive frailty

In line with the definition by an (I.A.N.A./I.A.G.G.) international consensus group [3], cognitive frailty was defined as the simultaneous presence of both cognitive impairment and physical frailty [3], and has been previously validated [41–43]. Based on the two components—cognitive impairment and physical frailty, we defined four combined groups:

Group 1: normal cognition and nonfrail

Group 2: cognitive impairment and nonfrail

Group 3: normal cognition and frail

Group 4: cognitive impairment and frail (cognitive frailty)

### Disability and hospitalization

We considered important patient-centered outcomes: disability and hospitalization, as we did before [23]. Three domains (BADL, IADL, and mobility) of disability were operationalized, including 5 BADL (dressing, bathing, eating, getting in/out of bed, and using the toilet), 5 IADL (managing money, taking medications, shopping for groceries, meal preparation, and cleaning house), and 7-item mobility activities (100m, climbing several flights of stairs, getting up from a chair, stooping or kneeling or crouching, extending arms up, lifting 11 lb, and picking up a small coin). For each task in the three domains, participants were asked, “Do you have difficulty in performing the task?” Those participants who need personal assistance in performing one or more of the corresponding activities in each domain were defined as disability. Hospitalization was derived from the participants’ self-report of whether they received any inpatient care in the past year. Since the timing of developing disability during the follow-up period was not available [44], we defined a binary outcome to denote the occurrence of disability over the 4-year follow-up. For hospitalization, we did a similar definition to denote whether the participant had hospitalization over the 4-year follow-up since baseline in this study.

### Death

The death information in CHARLS was collected from the exit interview in 2013 and 2015 waves. Generally, a binary variable representing occurrence of death and a variable representing date of death were provided. However, date of death was only available in the 2013 wave according to the current dataset in the CHARLS website. Therefore, we constructed a binary variable to denote occurrence of death within the 4-year follow-up since baseline in this study.

### Covariates

Demographic characteristics including age, gender, residence (urban vs rural), education, and marital status were collected at baseline. Five categories were considered for education: no formal education/illiterate, can read but did not finish elementary school, elementary school/traditional Chinese school, middle school, and high school or above. Marital status was defined as currently married (including partnered), and others (e.g., separated, divorced, widowed). Health behaviors included smoking status (never, ever, and currently smoking), alcohol consumption (yes/no), and body mass index (BMI, kg/m^2^).

Self-reported comorbidity included ten self-reported chronic diseases by asking “Have you been diagnosed with the following conditions by a doctor”: hypertension; diabetes or high blood sugar; cancer or malignant tumor; chronic lung disease; heart problems; stroke; kidney disease; stomach or other digestive disease; arthritis or rheumatism; and asthma. The total number of chronic diseases was calculated. Multimorbidity was defined as co-existence of two or more chronic diseases within one older adult [18].

### Statistical analyses

Characteristics of the study population were presented as means (± standard deviation [SD]) or frequencies (percentage) in the overall sample, and four combined groups (Group 1–4).

Logistic regression models were used to estimate the individual (i.e., cognitive impairment and physical frailty as independent categorical variable respectively) and combined associations (i.e., the I.A.N.A./I.A.G.G. criteria defined four cognitive frailty groups as independent categorical variable, with Group 1 as the reference group) of cognitive impairment and physical frailty with adverse health outcomes. Note that we ran a logistic regression model for each health outcome (e.g., BADL disability) in participants who did not have exposure for that outcome at baseline (e.g., n=3341 participants without BADL disability at baseline). We adjusted for age and gender in model 1 and presented the odds ratios (ORs) and corresponding 95% confidence interval (CI). We further adjusted for other covariates including residence, education, marital status, smoking status, alcohol consumption, BMI, and multimorbidity in model 2.

To evaluate whether the associations of cognitive frailty with health outcomes differed by multimorbidity, we added the interaction terms: the four cognitive frailty groups × multimorbidity in the model 1. We then repeated the above analyses in two subgroups: those with and without multimorbidity and presented the results separately.

Sensitivity analyses were performed to test the robustness of the results. First, as the information on dementia was not available in CHARLS, we used one item—self-reported memory related problem (dementia, Parkinson's disease, etc.) as a proxy for dementia. We excluded participants with a response of “Yes” to this question and rerun the model 1 above to testify whether the individual and combined associations of cognitive impairment and physical frailty with adverse health outcomes changed. Second, to address the concern about the attrition of study participants, particularly due to death, we used a competing risk Cox regression model [45] to re-examine the above individual and combined associations of cognitive impairment and physical frailty with disability and hospitalization. To do so, we assumed that the date of incurring disability, hospitalization, or death was the time of the survey if the participant reported having that disability, hospitalization, or death (exit interview). The follow-up time was calculated from the baseline to the date of incurring disability (hospitalization) or death, whichever came first. Finally, we ran an additional analysis by examining the cross-sectional association of cognitive impairment, physical frailty, and their combinations with the health outcomes, in order to confirm the findings from the majority of the literature on a cross-section study design.

All statistical analyses were performed using SAS version 9.4 (SAS Institute, Cary, NC). P<0.05 (two-tailed) was considered as statistically significant.

## Supplementary Material

Supplementary Tables
