# Supplementary material for: Cognitive frailty in relation to adverse health outcomes independent of multimorbidity: results from the China health and retirement longitudinal study
Source: Aging (Albany NY). 2020 Nov 18;12(22):23129–45. doi: 10.18632/aging.104078 (PMC7746379; doi:10.18632/aging.104078)
Supplement: Supplementary Tables [file aging-12-104078-s001..pdf]

SUPPLEMENTARY TABLES

Supplementary Table 1. Summary of the compatible studies mentioned in our study.

First author	Year	Country	N	Age	study types	Frailty criteria	Cognition	BADL	IADL	Mobility disability	Hospital ization	Mortality
Aliberti MJ[7]	2019	USA	7338	≥65	6 years longitudinal	Fried PF	HRS tests	↑	/	/	/	↑
Avila-Funes JA[8]	2009	French	6030	65-95	4 years longitudinal	Fried PF	MMSE	↑	↑	↑	↑	→
Feng L[9]	2017	Singapore	2375	≥55	6 years longitudinal	Fried PF	MMSE	↑	↑	/	→	↑
John PDS[10]	2017	Canada	1751	≥65	5 years longitudinal	FI	MMSE	/	/	/	/	↑
Cano C[11]	2012	USA	1815	≥67	10 years longitudinal	Fried PF	MMSE	/	/	/	/	→
Solfrizzi V[12]	2017	Italian	2373	65-84	4 years longitudinal	Fried PF	MMSE	/	/	/	/	↑
Solfrizzi V[13]	2017	Italian	2150	65-84	7 years longitudinal	Fried PF	MMSE	/	/	/	/	↑
Yu R[14]	2018	China	3491	≥65	12 years longitudinal	Fried PF	MMSE	/	/	↑	↑	↑
Shimada H[15]	2016	Japan	8864	≥65	cross-sectional	Fried PF	NCGG-FAT	/	↑	/	/	/
Z Liu[23]	2018	USA	754	≥70	11 years longitudinal	Fried PF	MMSE	↑	↑	↑	→	
Roppolo M[S1]	2016	Italian	594	≥65	cross-sectional	Fried PF	MMSE	/	/	↑	/	/

BADL, basic activity of daily living; IADL, instrumental activity of daily living; Fried PF, Fried physical frailty; FI, frailty index; MMSE, Mini-mental State Examination; NCGG-FAT, National Center for Geriatrics and Gerontology-Functional Assessment Tool; MCA, Montreal Cognitive Assessment.
↑, refers to cognitive frailty was associated with increased risk of adverse health outcomes; →, refers to no statistically significant association; /, refers to do not mention.
[7-15, 23] keep the same citation with Manuscript Text.

Supplementary Table 2. Mutually controlled associations of cognitive impairment and physical frailty with disability (BADL, IADL, and mobility), hospitalization, and death in full sample, CHARLS 2011-2015.

	BADL disability	IADL disability	Mobility disability	Hospitalization	Death
**Individual effect/** No.	3341	3226	1685	3776	5113
Cognition					
Normal cognition	Ref.	Ref.	Ref.	Ref.	Ref.
Cognitive impairment	1.39 (1.13–1.71)	2.10 (1.69–2.61)	1.53 (1.06–2.21)	1.13 (0.93–1.38)	1.53 (1.16–2.01)
Physical frailty					
Nonfrail	Ref.	Ref.	Ref.	Ref.	Ref.
Frail	2.34 (1.63–3.37)	2.39 (1.60–3.57)	1.52 (0.64–3.60)	1.36 (1.01–1.82)	2.01 (1.48–2.72)

CHARLS, the China Health and Retirement Longitudinal Study; BADL, basic activities of daily living; IADL, instrumental activities of daily living.
As described in Methods, we ran a logistic regression model for each health outcome (e.g., BADL disability) in participants who did not have exposure for that outcome at baseline (i.e., N=3341 participants without BADL disability at baseline). Odds ratios (ORs) and corresponding 95% confidence interval (CI) are presented. The model adjusted for age, gender, cognition, and physical frailty.

Supplementary Table 3. Associations of multimorbidity with disability (BADL, IADL, and mobility), hospitalization, and death in full sample, CHARLS 2011-2015.

	BADL disability	IADL disability	Mobility disability	Hospitalization	Death
Multimorbidity /No.	3341	3226	1685	3776	5113
No	Ref.	Ref.	Ref.	Ref.	Ref.
Yes	2.10 (1.81–2.44)	1.92 (1.65–2.23)	1.95 (1.54–2.47)	1.81 (1.57–2.09)	1.26 (1.03–1.54)

CHARLS, the China Health and Retirement Longitudinal Study; BADL, basic activities of daily living; IADL, instrumental activities of daily living.
As described in Methods, we ran a logistic regression model for each health outcome (e.g., BADL disability) in participants who did not have exposure for that outcome at baseline (i.e., N=3341 participants without BADL disability at baseline). Odds ratios (ORs) and corresponding 95% confidence interval (CI) are presented. The model adjusted for age and gender.

Supplementary Table 4. Associations of cognitive impairment and frailty with disability, hospitalization, and death in sample after removing those with memory problem, CHARLS 2011-2015.

	BADL disability	IADL disability	Mobility disability	Hospitalization	Death
**Individual effect**					
Cognition					
Normal cognition	Ref.	Ref.	Ref.	Ref.	Ref.
Cognitive impairment	1.39 (1.13–1.71)	2.11 (1.69–2.63)	1.54 (1.07–2.23)	1.11 (0.91–1.36)	1.57 (1.19–2.07)
**Physical frailty**					
Nonfrail	Ref.	Ref.	Ref.	Ref.	Ref.
Frail	2.34 (1.61–3.38)	2.48 (1.66–3.72)	1.46 (0.61–3.49)	1.38 (1.03–1.87)	2.05 (1.50–2.80)
**Combined effect**					
Normal cognition & Nonfrail	Ref.	Ref.	Ref.	Ref.	Ref.
Cognitive impairment & Nonfrail	1.40 (1.13–1.73)	2.12 (1.69–2.65)	1.52 (1.05–2.20)	1.15 (0.93–1.41)	1.41 (1.04–1.92)
Normal cognition & Frail	2.48 (1.64–3.73)	2.54 (1.65–3.92)	1.33 (0.54–3.22)	1.53 (1.09–2.15)	1.80 (1.25–2.60)
Cognitive impairment & Frail	2.32 (1.01–5.32)	4.13 (1.40–12.15)	1*	1.06 (0.56–2.02)	3.76 (2.14–6.61)

CHARLS, the China Health and Retirement Longitudinal Study; BADL, basic activities of daily living; IADL, instrumental activities of daily living.
As described in Methods, we ran a logistic regression model for each health outcome (e.g., BADL disability) in participants who did not have exposure for that outcome at baseline (i.e., N=3341 participants without BADL disability at baseline). Odds ratios (ORs) and corresponding 95% confidence interval (CI) are presented. The model adjusted for age and gender.
*Since all participants in this subgroup reported the health outcome over the follow-up period, we assigned 1 to them.

Supplementary Table 5. Associations of cognitive impairment and frailty with disability and hospitalization accounting for the competing risk of death, CHARLS 2011-2015

	BADL disability	IADL disability	Mobility disability	Hospitalization
Individual effect				
Cognition				
Normal cognition	Ref.	Ref.	Ref.	Ref.
Cognitive impairment	1.27 (1.10–1.47)	1.63 (1.42–1.86)	1.17 (1.02–1.34)	1.06 (0.86–1.32)
Physical frailty				
Nonfrail	Ref.	Ref.	Ref.	Ref.
Frail	1.57 (1.28–1.93)	1.82 (1.48–2.24)	1.17 (0.89–1.55)	1.40 (1.06–1.87)
Combined effect				
Normal cognition & Nonfrail	Ref.	Ref.	Ref.	Ref.
Cognitive impairment & Nonfrail	1.31 (1.13–1.52)	1.65 (1.44–1.90)	1.17 (1.02–1.34)	1.09 (0.87–1.36)

Normal cognition & Frail	1.75 (1.40–2.19)	1.92 (1.53–2.40)	1.16 (0.84–1.60)	1.49 (1.09–2.04)
Cognitive impairment & Frail	1.24 (0.77–2.01)	2.16 (1.32–3.52)	1.46 (1.10–1.94)	1.18 (0.62–2.24)

CHARLS, the China Health and Retirement Longitudinal Study; BADL, basic activities of daily living; IADL, instrumental activities of daily living.
As described in Methods, we ran a competing risk Cox regression model for each health outcome (e.g., BADL disability) in participants who did not have exposure for that outcome at baseline (i.e., N=3341 participants without BADL disability at baseline). Hazard ratios (HRs) and corresponding 95% confidence interval (CI) are presented. The model adjusted for age and gender.
*Since all participants in this subgroup reported the health outcome over the follow-up period, we assigned 1 to them.

Supplementary Table 6. Cross-sectional associations of cognitive impairment and frailty with disability and hospitalization at baseline, CHARLS 2011.

	BADL disability	IADL disability	Mobility disability	Hospitalization
No.	1130	1289	3199	602
**Individual effect**				
Cognition				
Normal cognition	Ref.	Ref.	Ref.	Ref.
Cognitive impairment	1.88 (1.58–2.22)	2.51 (2.14–2.96)	1.67 (1.41–1.99)	0.96 (0.75–1.22)
Physical frailty				
Nonfrail	Ref.	Ref.	Ref.	Ref.
Frail	3.83 (3.04–4.82)	4.58 (3.62–5.79)	5.03 (3.51–7.20)	1.61 (1.20–2.17)
**Combined effect**				
Normal cognition & Nonfrail	Ref.	Ref.	Ref.	Ref.
Cognitive impairment & Nonfrail	1.84 (1.53–2.21)	2.46 (2.07–2.92)	1.59 (1.33–1.90)	0.93 (0.72–1.21)
Normal cognition & Frail	3.90 (3.00–5.07)	4.53 (3.47–5.91)	4.57 (3.11–6.72)	1.60 (1.14–2.25)
Cognitive impairment & Frail	5.57 (3.55–8.74)	9.34 (5.66–15.42)	11.14 (4.06–30.57)	1.57 (0.87–2.82)

CHARLS, the China Health and Retirement Longitudinal Study; BADL, basic activities of daily living; IADL, instrumental activities of daily living.
As described in Methods, we ran a logistic regression model for each health outcome (e.g., BADL disability) at baseline. Odds ratios (ORs) and corresponding 95% confidence interval (CI) are presented. All models were adjusted for age and gender.

REFERENCE

1. Roppolo M, Mulasso A, Rabaglietti E. Cognitive Frailty in Italian Community-Dwelling Older Adults: Prevalence Rate and Its Association with Disability. J Nutr Health Aging. 2017; 21:631–636. https://doi.org/10.1007/s12603-016-0828-5 PMID:28537326
